# Quantification of Representative Ciguatoxins in the Pacific Using Quantitative Nuclear Magnetic Resonance Spectroscopy

**DOI:** 10.3390/md15100309

**Published:** 2017-10-12

**Authors:** Tsuyoshi Kato, Takeshi Yasumoto

**Affiliations:** Japan Food Research Laboratories, Tama Laboratory, Nagayama 6-11-10, Tama, Tokyo 206-0025, Japan; yasumotot@jfrl.or.jp

**Keywords:** ciguatoxin-1B, ciguatoxin-3C, qNMR

## Abstract

The absolute quantification of five toxins involved in ciguatera fish poisoning (CFP) in the Pacific was carried out by quantitative ^1^H-NMR. The targeted toxins were ciguatoxin-1B (CTX1B), 52-epi-54-deoxyciguatoxin-1B (epideoxyCTX1B), ciguatoxin-3C (CTX3C), 51-hydroxyciguatoxin-3C (51OHCTX3C), and ciguatoxin-4A (CTX4A). We first calibrated the residual protons of pyridine-*d*_5_ using certified reference material, 1,4-BTMSB-*d*_4_, prepared the toxin solutions with the calibrated pyridin-*d*_5_, measured the ^1^H-NMR spectra, and quantified the toxin using the calibrated residual protons as the internal standard. The absolute quantification was carried out by comparing the signal intensities between the selected protons of the target toxin and the residual protons of the calibrated pyridine-*d*_5_. The proton signals residing on the ciguatoxins (CTXs) to be used for quantification were carefully selected for those that were well separated from adjacent signals including impurities and that exhibited an effective intensity. To quantify CTX1B and its congeners, the olefin protons in the side chain were judged appropriate for use. The quantification was achievable with nano-molar solutions. The probable errors for uncertainty, calculated on respective toxins, ranged between 3% and 16%. The contamination of the precious toxins with nonvolatile internal standards was thus avoided. After the evaporation of pyridine-*d*_5_, the calibrated CTXs were ready for use as the reference standard in the quantitative analysis of ciguatoxins by LC/MS.

## 1. Introduction

**Figure 1:**
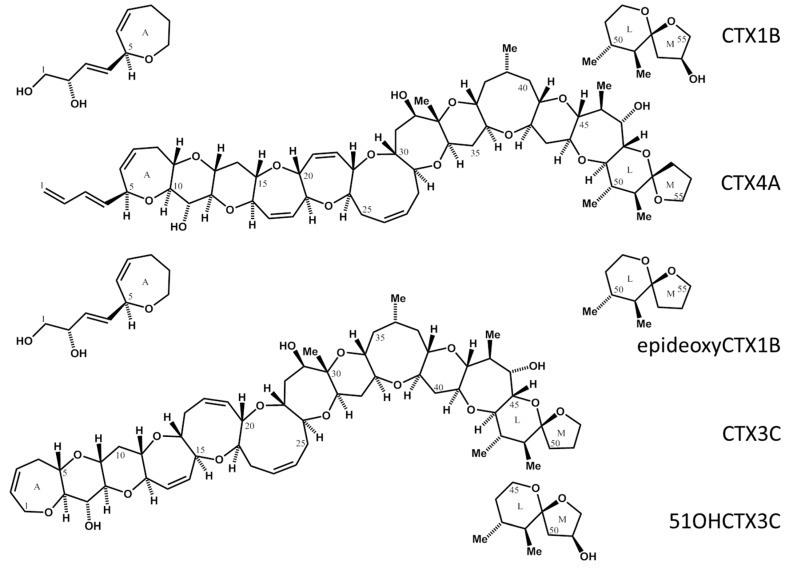
Structure of CTXs. CTX1B, Chemical Formula: C_60_H_86_O_19_, Molecular Weight: 1111.31; deoxyCTX1B, Chemical Formula: C_60_H_86_O_18_, Molecular Weight: 1095.31; CTX3C, Chemical Formula: C_57_H_82_O_16_, Molecular Weight: 1023.25; 51OHCTX3C, Chemical Formula: C_57_H_82_O_17_, Molecular Weight: 1039.25; CTX4A, Chemical Formula: C_60_H_84_O_16_, Molecular Weight: 1061.2994.

Ciguatera fish poisoning (CFP) refers to a peculiar form of neurologic poisoning resulting from the ingestion of fish inhabiting warm water regions. Though the mortality rate is low, the morbidity rate for CFP is the highest among the poisoning of natural etiology, with an estimated number of around 50,000 patients annually [1]. In the Pacific, the causative toxins, named ciguatoxins (CTXs), are produced by microalgae, *Gambierdiscus* spp., and accumulate in various species of fish via the food chain [2]. The toxicity of individual fish is unpredictable and markedly fluctuates, as the population of the causative alga and feeding history of fish can greatly vary [3]. To protect human health and avoid serious economic loss due to the implication of commercially important species, proper measures to detect toxins are highly desired. Mouse bioassays [4] have been the routine practice to detect CTXs since early days but need to be substituted by other methods of higher sensitivity and specificity, not to mention the call to limit animal use for routine assays. Previously, we proposed using an LC/MS method as an alternative and successfully revealed the details of regional and species variations of toxin profiles in fish and causative algae [5]. The method is highly sensitive and produces accurate data, but we have to clear three hurdles to promote its wide use. First, we have to quantify extremely low concentrations of the toxins. The consensus on the regulation level is 0.01 ppb for ciguatoxin 1B or its equivalent in flesh [6]. Even higher is the second hurdle to prepare pure toxins to be used as standards. The major toxin, ciguatoxin-1B, needed four tons of toxic fish collected over ten years to obtain 0.35 mg. Much effort had to be continued, therefore, to prepare toxins of less abundance. The final problem to be solved was how to quantify such minute samples. The toxins have no chromophore. Complete drying for weighing is defied because recovery from the vessel surface becomes difficult. Therefore, we chose a quantitative ^1^H-NMR (qNMR) method for quantitation [7]. The foreseeable problems in NMR were the small samples, multiple and often overlapping proton signals, the large ladder-shape structure comprising stereo-flexible medium size rings (seven, eight, and nine membered), and the large signals of water that remain in the samples. The routine practice of using Certified Reference Material (CRM) as an internal standard [8] was disfavored to avoid the contamination of the target toxins with the nonvolatile CRMs. Instead, we used CRM to quantify the residual protons in pyridine-*d*_5_ and subsequently used the calibrated pyridine-*d*_5_ as the secondary inner standard to quantify the CTXs. Ciguatoxins present in fish in the Pacific consist of two groups differing in their skeletal structures, ciguatotoxin-1B type, and ciguatoxin-3C type. Hence, the selection of suitable protons in each type is required for quantification. We carried out the NMR measurements, meticulously choosing the parameters. The quantity of each CTX was calculated by employing the signal area ratio accurately integrated between the CTXs and calibrated pyridine-*d*_5_ proton signals. Thus, we achieved, for the first time, the quantification of five toxins important in monitoring fish toxicity in the Pacific (Figure 1): CTX1B, epideoxyCTX1B, CTX3C, 51OHCTX3C, and CTX4A. The calibrated toxins will serve as an invaluable tool to identify and quantify the toxins in fish that have remained elusive in the past. A great contribution is expected to toxicology, epidemiology, environmental studies, and commercial fisheries.

## 2. Results

### 2.1. Determination of the Residual Proton Content of Pyridine-d_5_ and Purity Calculation

**Figure 2:**
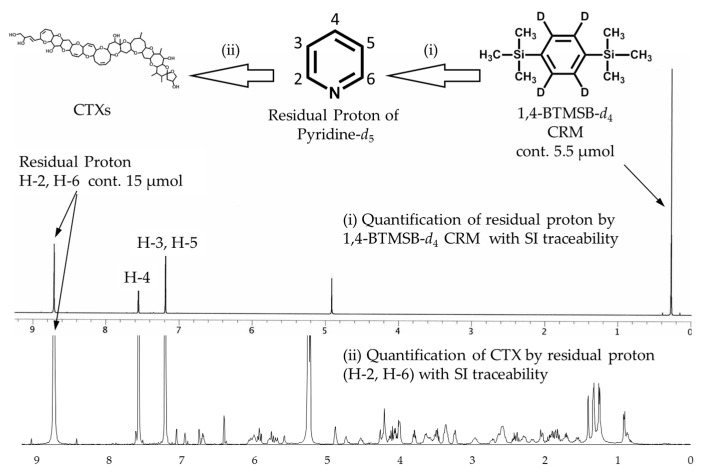
Scheme for the indirect determination of CTX and for keeping the traceability to International System of Units (SI) using qNMR by employing volatile substances as the internal standard. The upper spectrum exhibits the residual proton signals of pyridine-*d*_5_, and an internal standard signal of 1,4-BTMSB-*d*_4_. The lower spectrum exhibits signals of CTX and an internal standard signal of residual proton. The signal intensity of the residual proton contained in the CTXs/pyridine-*d*_5_ solution was approximately 30 when the signal of 1,4-BTMSB-*d*_4_ was taken as 100.

The ^1^H-NMR spectrum of pyridine-*d*_5_ (Figure 2) exhibited three residual proton signals arising from H-2 and H-6 (8.76 ppm), H-4 (7.61 ppm), and H-3 and H-5 (7.24 ppm). The residual proton content in pyridine-*d*_5_ was calculated by ^1^H-NMR experiments using 1,4-BTMSB-*d*_4_ as the internal standard, based on the following Equation (1):

(1) $$Value_{A}=\frac{I_{A}}{I_{IS}}\times \frac{N_{IS}}{N_{A}}\times \frac{M_{A}}{M_{IS}}\times \frac{W_{IS}}{W_{A}}\times P_{IS}$$

where *Value_A_* is the quantity of the residual proton in the pyridine-*d*_5_; *I_A_* is the area ratio of each individual (define ratio) signal arising from the residual proton; *I_IS_* is the area ratio of the internal standard; *N_IS_* is the number of protons of the internal standard; *N_A_* is the number of protons of the residual proton; *M_A_* is the mole weight of the residual proton, calculated by employing C_5_H_5_N as the chemical formula; *M_IS_* is the mole weight of the internal standard; *W_IS_* is the weight of the internal standard; *W_A_* is the weight of the pyridine-*d*_5_; and *P*_IS_ is the purity of the internal standard.

The quantity of each residual proton signal in pyridine-*d*_5_ calculated was determined to be 0.149%, 0.159%, and 0.153%, respectively. Those residual proton signals showed good repeatability (relative standard deviations (RSD) ranged from 0.30% to 0.33%) between the content of five ampoules, indicating the validity of the signals as internal standards. In the same production lot of ampoules, the residual proton of pyridine-*d*_5_ was stably included in a fixed amount. Thus, the use of the same lot of pyridine-*d*_5_ ampoules as an internal standard solution was enabled. On the other hand, the dispersion between three signals was as large as 3.1% RSD, and it was considered that the deuteration ratio differs for each position of pyridine. The residual proton amount was not averaged among the signals and was used as an individual value.

The pre-calibrated pyridine-*d*_5_ ensures an ability to trace an absolute quantity of the CTX standards to the SI units. Of the three signals of the residual protons, two signals at a higher field (7.61 and 7.24 ppm) were overlapped with the hydroxy protons of 11-OH and 47-OH in CTX1B or 7-OH and 44-OH of CTX3C and thus judged unsuitable for use as the internal standard. Being free of contamination, the signal at 8.43 ppm (H-2, H-6) was judged appropriate for calculation (Figure 2).

### 2.2. Quantification of Respective CTXs

**Figure 3:**
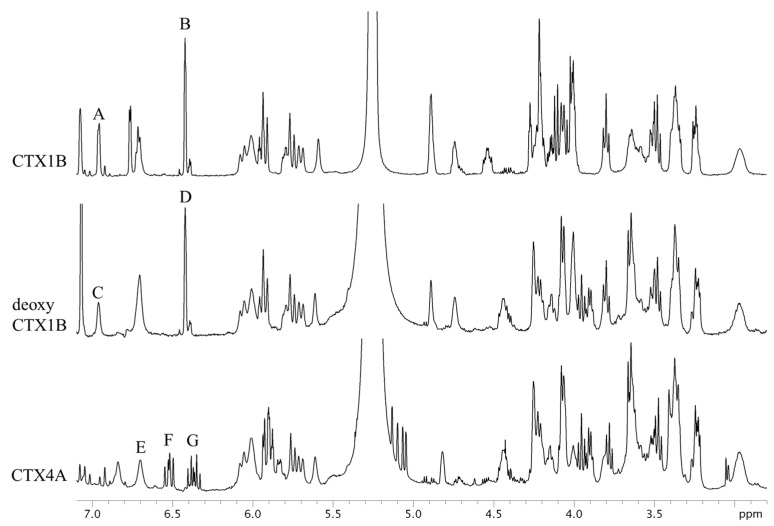
The ^1^H-NMR spectra of CTX1B, epideoxyCTX1B, and CTX4A. The parameters and conditions for the measurements are set as follows: CTX1B, instrument; Varian NMR System 500; data point, 65,536; pre scans, two times; number of scans, 512 times; relaxation delay, 60 s; temperature, 5 °C; acquisition time, 4 s. For epideoxyCTX1B; instrument, Varian NMR System 500; data point, 65,536; pre scans, two times; number of scans, 2048 times; relaxation delay, 60 s; temperature, 5 °C; acquisition time, 4 s. For CTX4A; instrument, Varian NMR System 500; data point, 65,536; pre scans, two times; number of scans, 3072 times; relaxation delay, 60 s; temperature, 5 °C; acquisition time, 4 s.

The ^1^H-NMR spectra of CTX1B, epideoxyCTX1B, and CTX4A are shown in Figure 3. The signals, though congested, were assignable based on the reference [9,10]. Apparently, the signals suitable for quantitative use are limited to a few arising from the protons of olefins and hydroxyls. They are moderately well separated from the congested signals of the protons on the skeletal structures. The separation between the olefin and hydroxyl proton signals was best achieved by measuring at 5 °C. The signals B and D, characteristic to the 3-butene-diol side-chain of the CTX1B and epideoxyCTX1B, feature narrow line widths and sufficient intensity to make them suitable for calculation. Calculations based on these signals placed the content at 0.14 mg (0.2% RSD) for CTX1B and 0.06 mg (0.5% RSD) for epideoxyCTX1B. Similarly, signals A and C, arising from 2-OH located in the lower-magnetic field, had narrow line widths and adequate intensity. The CTX content calculated therefrom was almost a complete match with those from the aforementioned signals B and D. The accuracy of the quantification was thus supported (Table 1). The protons that produced signals A, B, C, and D do not belong to the polyether rings but reside on the side chains. Unlike the protons on the cyclic rings, the protons on the side-chain are less susceptible to the conformational changes of the skeletal rings and, therefore, the broadening of the signals is reduced. The characteristic multiplet signals, F and G, arising from the 1,3-diene of the side chain in CTX4A also feature narrow line widths suitable for the CTX4A quantification. However, the multiple line shape resulted in low signal intensity and inevitably led to a large dispersion of the quantitative value (0.07 mg; 2.3% RSD), as compared with the value obtained on an equivalent concentration of epideoxyCTX1B (0.06 mg; 0.5% RSD).

**Figure 4:**
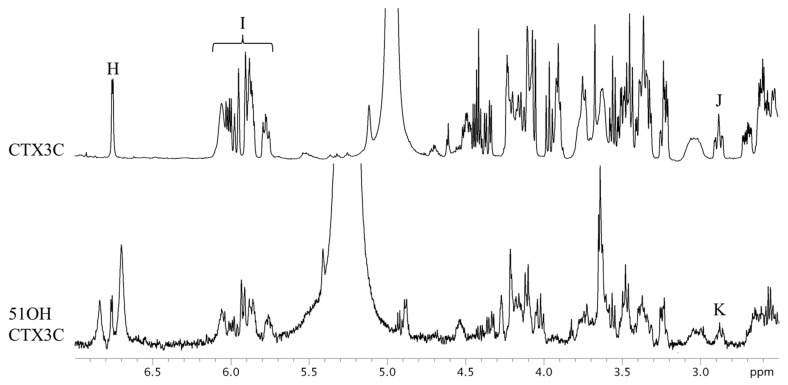
The ^1^H-NMR spectra of CTX3C and 51OHCTX3C. The parameters and conditions for the measurements are given below. CTX3C: Instrument, Varian NMR System 500; data point, 65,536; pre scans, two times; number of scans, 512 times; Relaxation delay, 60 s; temperature, 25 °C; Acquisition time, 4 s. 51OHCTX3C: Instrument, Varian NMR System 500; data point, 65,536; pre scans, two times; number of scans, 1280 times; Relaxation delay, 60 s; temperature, 5 °C; Acquisition time, 4 s.

**Figure 5:**
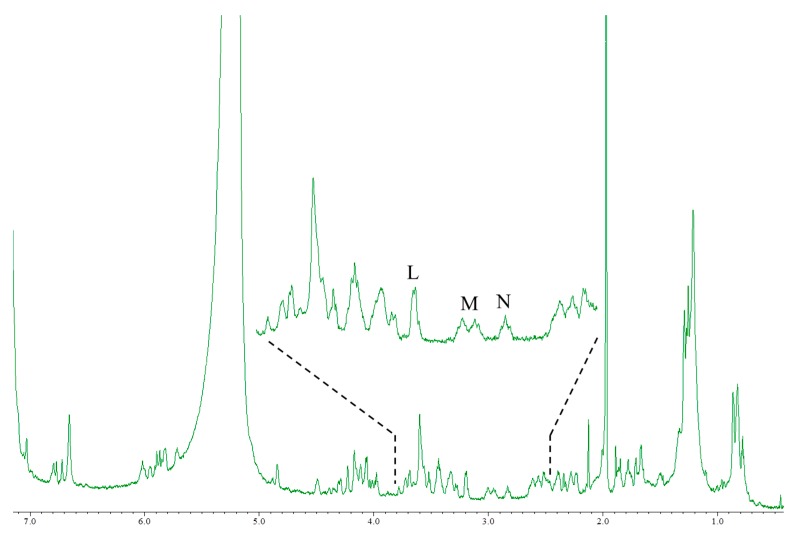
The ^1^H-NMR spectrum of 51OHCTX3C using high magnetic field NMR. Instrument, JNM-ECA800 with cryogenically cooled probe (Jeol Ltd., Tokyo, Japan); irradiation frequency, 800.14 MHz; data point, 65,536; pre scans, 32 times; number of scans, 512 times; Relaxation delay, 56.7 s; temperature, 5 °C; Acquisition time, 3.3 s.

CTX3C and 51OHCTX3C lack the side chain useful for the quantification of the aforementioned CTXs. All the protons were tested for suitability for quantitative determination (Table 2). The CTX3C content was calculated to be 0.41 mg (0.9% RSD) using the signal H (44-OH), as shown in Figure 4. The hydrogen-bond formation of hydroxyl protons gave rise to a wide variety of line shapes. Nevertheless, the signals were narrow and sufficiently strong and produced values more accurate than those obtained from other signals (I and J). Since the 44-OH signal of 51OHCTX3C was observed to have significant overlaps with impurities, the calculation was performed using the methylene proton signals K (H-17) positioned at 2.9 ppm. The amount of 51OHCTX3C was calculated to be 0.013 mg (1.5% RSD), but we concluded that the 500-MHz NMR instrument does not provide enough sensitivity to the small sample of only one-tenth the quantity of CTX1B. Therefore, we carried out measurements using an 800-MHz NMR instrument equipped with a cryogenically-cooled probe. The quantities of 51OHCTX3C could be determined down to 0.011 mg using the amplified signals L (H-38, 42), M (H-22, 25), and N (H-17), as shown in Figure 5.

## 3. Discussion

Since ciguatera fish poisoning is the largest category of food poisoning of natural etiology, it has stimulated scientists to test and propose various testing methods. The use of anti-toxin antibodies faces problems arising from the abundance of structurally variant congeners. The functional assay based on the specific binding of the toxins to the voltage-dependent sodium channel uses a radio-active ligand, which requires strict regulatory control. The function based cytotoxicity assay provides the highest sensitivity. However, the practical merit of the method awaits future validation on various fish species. The rapid progress in LC/MS analysis promises its potential due to its sensitivity and accuracy. In all analytical methods, including LC/MS, the use of a reliable standard is imperative. Nevertheless, the preparation of CTXs standards is a great challenge for two reasons. First, the availability of CTXs is extremely limited because most CTXs are the metabolites of fish and unavailable in algal cultures. The second problem is related to the technology itself. Containing 82 to 86 protons, CTXs produce congested and partially overlapped NMR signals. Many flexible rings in the structure allow multiple conformers that lead to the broadening of signals. Despite these difficulties, the qNMR method successfully achieved the quantification of CTX1B, epideoxyCTX1B, CTX4A, CTX3C, and 51OHCTX3C by choosing the proper signals. The olefinic protons arising from side chains were preferred to those on the polyether rings to avoid broadened signals, enabling quantification down to 0.06 mg (deoxyCTX1B) with high accuracy. The absence of the side-chain in CTX3C and its analogues made us newly select other protons. After vigorous testing of every signal, we found the signals arising from 44-OH and H-17 to possess the necessary quality for qNMR. The quantity of 51OHCTX3C amounted to only 1/40 of CTX3C, which was too small to produce valid signals on a 500-MHz instrument. The high field 800-MHz NMR instrument equipped with a cryogenically-cooled probe could quantify CTXs down to 0.01 mg. CTXs thus quantified are planned to be used as standards in LC/MS measurements. 

After the measurement of the NMR spectra, each CTX reference standard solution was individually prepared by diluting the CTX test solution with methanol. The CTX reference standard solution was dispensed in small aliquots into small glass vials, and the solvent was removed by a drying operation. To prevent the nonspecific adsorption of CTXs onto the glass wall surface, a very small amount of ethanol was added to each vial.

## 4. Materials and Methods

### 4.1. Materials

CTX1B, CTX3C, CTX4A, eipdeoxyCTX1B, and C51OHCTX3C used in previous structure-works were used. NMR test tubes with 5 mm outer diameters were purchased from Kusano Science Co. (Tokyo, Japan). Pyridine-*d*_5_ (99.8% deuterium content) was obtained from Merck KGaA (Darmstadt, Germany), and the CRM,1,4-bis(trimethylsilyl)benzene-*d*_4_ (1,4-BTMSB-*d*_4_, purity 99.8%, expanded uncertainty was 0.5%, *k* = 2), was from Wako Pure Chemical Industries, Ltd. (Osaka, Japan).

### 4.2. Apparatus

The experimental set up was composed of a semi-micro balance (AG285 or MS205DU, Mettler-Toledo, Greifensee, Switzerland), an ultra-micro balance (MSE2.7S, Sartorius AG, Göttingen, Germany), and an NMR spectrometer equipped with a Varian 5-mm indirect probe (Varian NMR System 500, Varian Technologies, Palo Alto, CA, USA).

### 4.3. Determination of the Residual Proton Content in Pyridine-d_5_

Accurately weighed 1,4-BTMSB-*d*_4_ CRM (1.0197–1.5668 mg) was mixed with pyridine-*d*_5_ (0.77421–0.80378 g) to afford test solutions of 1,4-BTMSB-*d*_4_ CRM (*N* = 5). The accurate quantity of pyridine-*d*_5_ was pre-determined by weighing the ampule before and after taking out pyridine-*d*_5_. The residual protons of pyridine-*d*_5_ were quantified by comparing the signal intensity with that of CRM.

### 4.4. Preparation of Test Solutions of CTX

CTX1B was dissolved in an ampule of pyridine-*d*_5_ to afford test solutions of CTX1B. The accurate amount of pyridine-*d*_5_ (0.79830 g) used for the test solution was determined by weighing with a semi-micro balance. The other CTXs were dissolved in 1 mL of pyridine-*d*_5_ to afford test solutions of CTX. The accurate amount of pyridine-*d*_5_ (1.04616–1.05397 g) used for each test solution was determined by weighing with a semi-micro balance. A 600 µL portion of the test solution was transferred into a NMR test tube for the measurement of the ^1^H-NMR spectrum.

### 4.5. ^1^H-NMR Measurements

The relaxation delay was set at six times the longest relaxation time (*T*_1_) of the pyridine signals to recover over 99% of z-magnetization [7]; *T*_1_ was pre-determined by an inversion-recovery test. The following settings were used for the qNMR experiments: irradiation frequency, 499.87 MHz; acquisition time, 4 s; relaxation delay, 60 s; probe temperature, 5 or 25 °C; spectral width, 40 ppm; FID data points, 161,290; number of scans, eight to 2560; spinning, off; dummy scans, two times; ^13^C decoupling, MPF8; pulse angle, 90°; pulse width, 10.4 µs.

### 4.6. Data Processing

**Figure 6:**
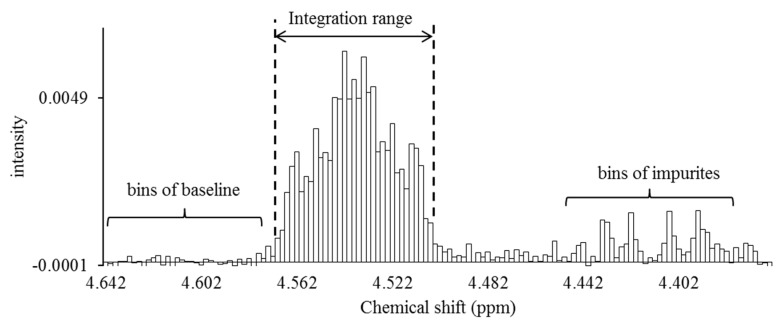
Signal integration using the spectral bucketing technique. All spectra were integrated as 0.002-ppm-sized buckets. The area ratio of each signal was calculated by adding all buckets larger than the buckets arising from the baseline. Baseline correction of all spectra was performed by using a software function before performing the integration processing. The area ratio of the baseline bucket is desirably close to zero after software baseline correction. If the baseline bucket had a relatively large area ratio, a correction was made by subtracting the area from all buckets to serve as a more precise baseline correction.

The data was processed with VnmrJ software ver. 2.3, supplied by the manufacturer (Varian Technologies, Palo Alto, CA, USA). Fourier transformation was performed on 262,144 data points without using window functions. All proton chemical shifts were referenced to a residual proton signal at positions 2 and 6 in pyridine-*d*_5_ at 8.765 ppm. The phase of all spectra was collected manually while observing a spectral line shape. The baseline of spectra was adjusted horizontality, and each signal area ratio was calculated by software functions. An integration range was individually optimized based on the line width of the signals and the space between the signals. The spectra were integrated using the spectral bucketing technique with 0.002-ppm-sized buckets [11]. The area ratio of each signal was calculated by adding all buckets larger than the buckets arising from the baseline (noise level; Figure 6).

### 4.7. Calculation of CTXs Content

The quantity of CTXs was calculated by ^1^H-NMR experiments using the residual proton signal of pyridine-*d*_5_ as the internal standard, based on the following Equation (2):

(2) $$Value_{A}=\frac{I_{A}}{I_{IS}}\times \frac{N_{IS}}{N_{A}}\times \frac{M_{A}}{M_{IS}}\times W_{IS}\times Q_{IS}$$

where *Value_A_* is the quantity of CTX (weight); *I_A_* is the area ratio of each individual signal arising from CTX; *I_IS_* is the area ratio of the residual proton of pyridine-*d*_5_; *N_IS_* is the number of protons of the residual proton of pyridine-*d*_5_; *N_A_* is the number of protons of CTX; *M_A_* is the mole weight of CTX; *M_IS_* is the mole weight of the residual proton of pyridine-*d*_5_; *W_IS_* is the weight of pyridine-*d*_5_; and *Q_IS_* is the quantity of the residual proton of pyridine-*d*_5_.

## 5. Conclusions

Five ciguatoxins represented in fish from the Pacific were quantified by ^1^H-NMR to be used in a quantitative analysis of ciguateric fish by LC/MS. Pyridine-*d*_5_ with predetermined residual protons was used to prepare test solutions and to exploit the residual protons as the inner standard. The following protons were selected for quantitation: CTX1B and epideoxyCTX1B, the olefinic protons on the side-chain; CTX3C, the 44-OH proton and all olefinic protons; 51OHCTX3C, 17-CH, 22-CH, 25-CH, 38-CH, and 42-CH; CTX4A, the olefinic protons of the side-chain and 47-OH. Quantification was possible with samples ranging from 0.01 to 0.4 mg. The conventional use of non-volatile reference material was avoided by the use of residual protons in the solvent of the test solution.

The CTXs’ reference standard was dispensed in small aliquots into small glass vials, and the solvent was removed by a drying operation. To prevent the nonspecific adsorption of CTXs onto the glass wall surface, a very small amount of ethanol was added to each vial.

## Figures and Tables

**Table 1:** The quantities of CTX1B, deoxyCTX1B, and CTX4A.

Compounds	Signals	Assign of Signals	S/N	Signal Intensity	Quantitative Value (mg)
CTX1B	A	Hydroxyl group 2	28	-	0.1374 ± 0.0012
	B	Olefin 3, 4	68	0.25	0.1381 ± 0.0003
deoxyCTX1B	C	Hydroxyl group 2	11	-	0.0590 ± 0.0017
	D	Olefin 3, 4	48	0.11	0.0586 ± 0.0003
CTX4A	E	Hydroxyl group 47	14	-	0.0832 ± 0.0007
	F	1,3-diene 3	20	0.075	0.0794 ± 0.0018
	G	1,3-diene 2	20	-	0.0743 ± 0.0017

The quantitative values were calculated using signals A to G in Figure 3. The signal intensity was expressed as a signal of 1,4-BTMSB-*d*_4_ as 100. Signals B of CTX1B, D of deoxyCTX1B, and F and G of CTX4A were used for the final quantification.

**Table 2:** The quantities of CTX3C and 51OHCTX3C.

Compounds	Signals	Assign of Signals	S/N	Signal Intensity	Quantitative Value (mg)
CTX3C	H	Hydroxyl group 44	81	0.40	0.4060 ± 0.0038
	I	Olefin 2, 3, 13, 14, 18, 19, 23, 24	-	-	0.4408 ± 0.0018
	J	17-H	37	-	0.4243 ± 0.0067
51OHCTX3C	K	17-H	<10	0.013	0.0134 ± 0.0002
	L	38, 42-H	25	-	0.01089
	M	22, 25-H	10	-	0.01230
	N	17-H	10	-	0.01085

The quantitative values were calculated using the signals H to K in Figure 4 and Lt o N in Figure 5. The signal intensity was expressed as a signal of 1,4-BTMSB-*d*_4_ as 100. Signals H of CTX3C and L to N of 51OHCTX3C were used for the final quantification.
